# DNA damage checkpoint execution and the rules of its disengagement

**DOI:** 10.3389/fcell.2022.1020643

**Published:** 2022-10-06

**Authors:** Candice Qiu Xia Yam, Hong Hwa Lim, Uttam Surana

**Affiliations:** ^1^ A*STAR Singapore Immunology Network, Singapore, Singapore; ^2^ Institute of Molecular and Cell Biology, Agency for Science, Technology and Research (A*STAR), Singapore, Singapore; ^3^ Department of Pharmacology, National University of Singapore, Singapore, Singapore

**Keywords:** cell division, mitosis, checkpoint, DNA damage, adaptation to chromosome damage

## Abstract

Chromosomes are susceptible to damage during their duplication and segregation or when exposed to genotoxic stresses. Left uncorrected, these lesions can result in genomic instability, leading to cells’ diminished fitness, unbridled proliferation or death. To prevent such fates, checkpoint controls transiently halt cell cycle progression to allow time for the implementation of corrective measures. Prominent among these is the DNA damage checkpoint which operates at G2/M transition to ensure that cells with damaged chromosomes do not enter the mitotic phase. The execution and maintenance of cell cycle arrest are essential aspects of G2/M checkpoint and have been studied in detail. Equally critical is cells’ ability to switch-off the checkpoint controls after a successful completion of corrective actions and to recommence cell cycle progression. Interestingly, when corrective measures fail, cells can mount an unusual cellular response, termed adaptation, where they escape checkpoint arrest and resume cell cycle progression with damaged chromosomes at the cost of genome instability or even death. Here, we discuss the DNA damage checkpoint, the mitotic networks it inhibits to prevent segregation of damaged chromosomes and the strategies cells employ to quench the checkpoint controls to override the G2/M arrest.

## Introduction

G1, S, and G2 phases, collectively known as interphase, account for the major portion of the division cycle. G2 phase, though much shorter than G1 and S phases, is an important period in the life of a dividing cell. It not only marks the completion of S phase, but it is also the gateway to mitosis when a cell “prepares’” for a dramatic upheaval in its internal organization. Chromosome condensation, nuclear membrane breakdown, Golgi fragmentation, mitotic spindle assembly, partitioning of duplicated chromosomes and cellular fission collectively represent intracellular organization in a dynamics flux. Soon, the “storm” passes and the progenitor cell undergoes self-cleavage, giving birth to two daughter cells with intracellular organization returning to its stable, interphase state. For the “preparation for M phase,” various networks pertaining to mitosis are primed (set in a ready-to-go state) in G2 such that all mitotic events are executed in a highly coordinated fashion. Cells that leave G2 phase and enter mitosis prematurely, face uncoordinated passage through M phase, resulting in genomic instability, reduced fitness or death (mitotic catastrophe) (Mc Gee, 2015). The length of G2 varies substantially among different organisms. Unlike vertebrate cells or fission yeast, the G2 phase in the budding yeast *Saccharomyces cerevisiae* is very brief or nonexistent. In *Xenopus laevis*, early embryonic divisions (and in other animal embryos) occur in rapid succession with an apparent omission of G1 and G2 (Siefert et al., 2015). Since mitotic event during these divisions are still executed in a coordinated fashion despite an apparent absence of G2, it suggests that the preparation for mitosis in these division formats begins before the completion of S phase or that there is a nearly complete overlap between the trailing part of S phase and G2.

G2 phase also serves as a “holding room” in the event cells incur DNA damage during S phase. Such damages result in the activation of the “DNA damage checkpoint control,” which halts the damaged cells in G2 and prevents them from executing M phase until the damage is fully repaired (Calonge and O’Connell, 2008; Ciccia and Elledge, 2010). In eukaryotes, two major modes of control are used to enact this blockade: by inhibiting CDK1 activation (i.e., onset of M phase) and/or by suppressing chromosome segregation. Once the DNA damage is successfully repaired, cells must disengage the mitotic machinery from the checkpoint control and proceed to mitosis (recovery). Intriguingly, when cells fail to repair the DNA damage, the checkpoint-mitosis disengagement can still occur after a pronged period of arrest and cells enter M phase with damaged chromosomes (adaptation). In this review, we discuss our current understanding of the main mechanisms underlying the activation of DNA damage checkpoint and its deactivation during recovery and adaptation. To set the context, we first briefly describe the mitotic networks, DNA damage checkpoint pathway and the nodes of “contact” between them.

## G2-TO-MITOSIS transition and CDK1/CYCLIN B kinase complex

**FIGURE 1:**
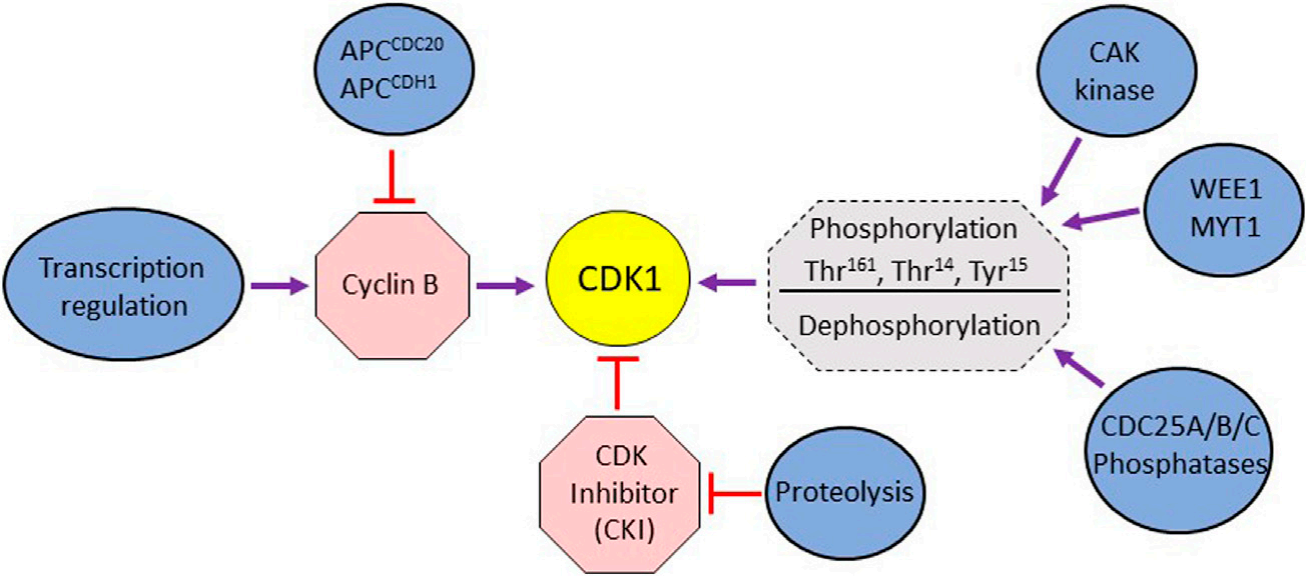
**(A)** Schematic representation of different modes of cellular regulation influencing mitotic activity of CDK1. The activation of CDK1 is directly regulated by cyclin B binding, phosphorylation by WEE1/MYT1 kinases, dephosphorylation by Cdc25 phosphatases and the binding of CDK inhibitors. CDK1’s activity is also indirectly affected by transcription regulation impinging on cyclin expression and the proteolytic degradation of cyclins and CDK inhibitors.

In vertebrate cells, G2/M DNA damage checkpoint halts cell cycle progression predominantly by inhibiting the regulatory network responsible for the entry into mitosis. The master regulator of the G2-to-M transition is the serine/threonine kinase complex CDK1/Cyclin B. The activity of CDK1 is governed primarily by its timely association with cyclin B. While the levels of CDK1 remain stable throughout the cell cycle, the Cyclin B levels fluctuate, reaching their highest during early mitosis and lowest at the end of M phase (Castedo et al., 2002; Sanchez et al., 2003). Cyclin B abundance is regulated at the transcriptional level as well as by proteolysis (Fung and Poon, 2005) (Figure 1). In vertebrates, transcription of Cyclin B is initiated in S phase and peaks in G2 and it is under the control of transcription factors NF-Y, FOXM1 and B-MYB (Lindqvist et al., 2009). Cyclin proteolysis starts during metaphase and continues throughout G1 (Bastians et al., 1999). The proteolytic degradation of Cyclin B is essential for cells’ exit from M phase and is mediated by the E3 ubiquitin ligase APC (anaphase promoting complex) (van Leuken et al., 2008). Since many substrates of CDK1/Cyclin B are nuclear proteins, the regulation of cellular localization of Cyclin B is also important for its association with CDK1. During interphase, Cyclin B is actively exported from the nucleus in the export-protein CRM1 dependent manner (Yang et al., 1998). CDK1 and PLK1 have been shown to phosphorylate Cyclin B at the CRM1 binding site, causing cyclin B’s net influx into the nucleus (Gavet and Pines, 2010).

The association of CDK1 and cyclin B is stabilized by phosphorylation of Thr^161^ (in human cells; Thr^167^ and Thr^169^ in *Schizosaccharomyces pombe* and *S cerevisiae*, respectively) in the T-loop of CDK1 by CAK kinase (CDK activating kinase) (Ducommun et al., 1991; Tassan et al., 1994; Dalal et al., 2004) (Figure 1). The activity of stable CDK1/cyclin B is negatively regulated by WEE1 kinase family (WEE1 and MYT1) that phosphorylates CDK1 on Thr^14^ and Tyr^15^ residues in the ATP binding site (Schmidt et al., 2017; Moiseeva et al., 2019) (Figure 1). WEE1 mediated phosphorylation holds CDK1 in an inactive state during G2 phase. At the onset of mitosis, the CDC25 family of phosphatases (CDC25A, B, and C in mammalian cells) reverses this inactivation by dephosphorylation of these residues, thereby activating CDK1/cyclin complex. Though CDC25A is predominantly nuclear, the CDC25B isoform shuttle between nucleus and cytoplasm during G2, (Lindqvist et al., 2005; Calonge and O'Connell, 2008; Moiseeva et al., 2019). The cytoplasmic localization of CDC25C during interphase is dependent on its binding to 14-3-3 protein which requires phosphorylation of CDC25C on S^216^ and S^287^ (Dalal et al., 2004). The nuclear translocation of CDC25C is facilitated by phosphorylation of the S^191^ and S^198^ residues (Toyoshima-Morimoto et al., 2002: Bahassi el et al., 2004). Active CDK1/cyclin B complex stabilizes CDC25A, prevents nuclear export of CDC25B and activates CDC25C (Kousholt et al., 2012). Together, the WEE1 kinase family (WEE1 and MYT1) and CDC25 phosphatase family constitute an ON/OFF switch for the CDK1/cyclin B activity (Figure 1). Active Cdk1/cyclin B also phosphorylates WEE1 and MYT1, causing their inactivation (Deibler and Kirschner, 2010). Thus, CDK1/cyclin B-mediated inactivation of WEE1/MYT1 and stabilization of CDC25 phosphatase family sets up a positive feedback loop that helps to amplify its own activity that peaks ∼30 min before prometaphase (Potapova et al., 2011). In mammalian cells, polo-like kinase PLK1 also helps to promote entry into mitosis. CDK1-mediated phosphorylation of WEE1 primes it for further phosphorylation by PLK1, thus aiding its inactivation (Ovejero et al., 2012; Parrilla et al., 2016). PLK1 can also activate the transcription factor FOXM1 involved in the expression of CDC25B (Mukhopadhyay et al., 2017). The activity of CDK1 is also regulated by cyclin-dependent kinase inhibitors (CKIs) under certain cellular contexts (Bunz et al., 1998; Satyanarayana et al., 2008).

While finer details of the G2-M transition may differ, the core aspects of CDK1/Cyclin B regulation by phosphorylation are highly conserved in lower eukaryotes such as yeasts *S. pombe* and *S. cerevisiae*. The onset of mitosis in these yeasts is regulated by cdc2 (cdk1)-cdc13 and Cdk1-Clb complexes, respectively (Li et al., 2009; Enserink and Kolodner, 2010). Interestingly, unlike *S. pombe* and mammalian cells, dephosphorylation of Tyr^15^ (Tyr^19^ in *S. cerevisiae*) is not a rate limiting step in *S. cerevisiae* in that the substitution of Tyr^19^ by alanine or inactivation of Swe1 (wee1 equivalent in *S. cerevisiae*) does not lead to premature onset of mitosis (Amon et al., 1992; Booher et al., 1993: Dalal et al., 2004). It is noteworthy that the critical involvement of Cdk1, wee1, APC and other effectors in the regulation of mitotic events was first discovered in these yeasts.

## Sister chromatid cohesion and chromosome segregation

**FIGURE 2:**
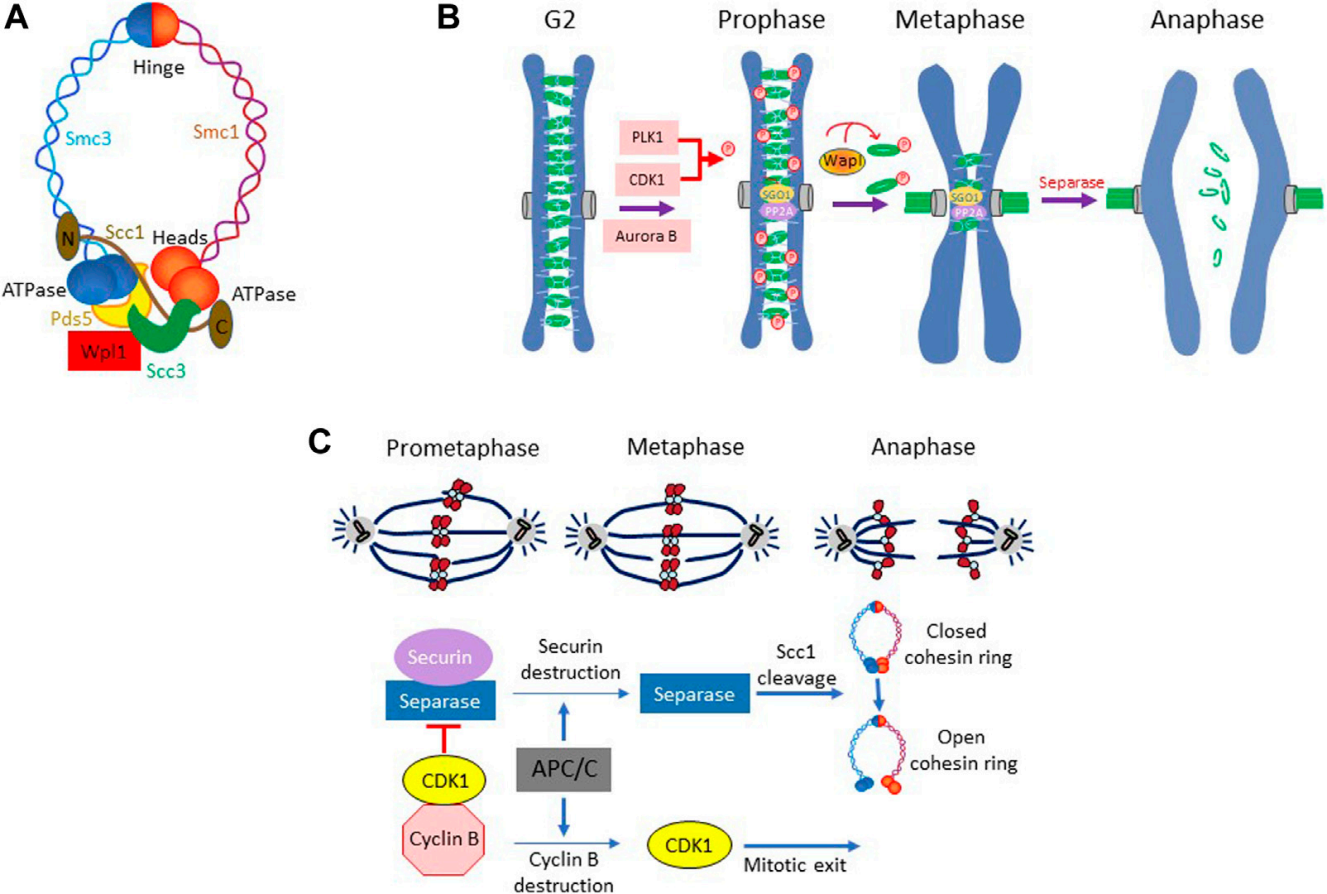
**(A)** The cohesion ring complex and its components in yeast *Saccharomyces cerevisiae*. **(B)** Prophase pathway in metazoan. In metazoans, chromosome arms cohesins are removed during prophase, while centromeric cohesins are protected by SGO1 and PP2A from removal and persists until the onset of anaphase. The removal of the arm-cohesins involves kinase activities of PLK1, Aurora B and CDK1 and the phosphorylation of cohesion subunit SA1 and SA2. In addition, WAPL plays an important role in coordinating cohesion removal during prophase. **(C)** Sister chromatid separation in *S. cerevisiae*. The cohesin complexes in *S cerevisiae* remain in place along the entire length of chromosome until the onset of anaphase. Dissolution of sister chromatid cohesion begins with the proteolytic degradation of securin Pds1 followed by separase mediated cleavage of cohesion subunit Scc1.

The yeast *S. cerevisiae* has been instrumental in the dissection of the DNA damage checkpoint pathway. However, unlike vertebrates, the checkpoint pathway in this organism does not target the events leading up to the onset of mitosis; instead, it inhibits the regulatory network that catalyzes sister chromatid segregation. Following chromosome duplication, the sister chromatids remain associated with each other until they are segregated away during anaphase due to the poleward pull exerted by the mitotic spindle. The cohesion between sister chromatids is mediated by cohesin complex. First reported in *S. cerevisiae*, it is composed of two SMC (Structural Maintenance of Chromosomes) proteins Smc1/Smc3 (Smc1α and Smc3 in human) and a kleisin subunit Scc1 (human Rad21), forming a ring-like arrangement (Makrantoni and Marston, 2018) (Figure 2A). The accessory proteins Scc3, Wpl1, and Pds5 (human SA1/SA2, Wapl, Pds5a/Pds5b) interact with Scc1 and regulate the association of the cohesin complex with the chromatin (Haering et al., 2002; Makrantoni and Marston, 2018). Loading of cohesins occurs prior to replication and is mediated by the Scc2-Scc4 loader. Stabilization of the cohesion complex requires entrapment of both sister chromatids and closing of the cohesin ring (Peters and Nishiyama, 2012). Subsequently, the ring is closed by Eco1 (ESCO1 and ESCO2 in human)-dependent acetylation of Smc3 head on Lys^112^ and Lys^113^, which prevents the DNA-stimulated ATP hydrolysis and inhibits the opening of the ring (Litwin et al., 2018). The sister chromatid cohesion in yeast is maintained along the entire length of the chromosome until the onset of anaphase (Marston, 2014). In mammalian cells, however, chromosome arm cohesins are removed during prophase (“prophase pathway”) in a CDK1-PLK1-dependent manner; only centromeric cohesins (protected by Shugoshin SGO1 and protein phosphatase 2A) persist until the onset of anaphase, giving metaphase chromosome their characteristic X-shape (McGuinness et al., 2005; Haarhuis et al., 2014) (Figure 2B).

At anaphase, sister chromatid cohesion must be dissolved to allow spindle to progressively partition the chromosomes to the opposite poles. In *S. cerevisiae*, the dissolution of cohesion is accomplished by abrupt opening of the cohesin ring by the protease Esp1 (separase or ESPL1 in human) which cleaves the cohesin subunit Scc1, allowing coordinated movement of chromosomes to the opposite poles (Luo and Tong, 2021) (Figure 2C). However, Esp1 remains in an inactive form due its association with securin Pds1 (PTTG in human) until the onset of anaphase (Mei et al., 2001; Han and Poon, 2013). Once all chromosomes are appropriately loaded onto the spindle, anaphase is triggered by APC^Cdc20^ mediated proteolytic degradation of securin Pds1 and releasing of separase Esp1 from Pds1-mediated inhibition (Shirayama et al., 1999; Mei et al., 2001) (Figure 2C). Esp1 then cleaves the cohesin subunit Scc1 at the core motif (D/E)-xxR and opens the cohesin ring, allowing spindle-powered poleward movement of sister chromatids (Sullivan et al., 2004; Zhang and Pati, 2017). This regulatory scheme that governs cohesion maintenance and its dissolution is highly conserved between yeast and vertebrates.

## G2/M transition under surveillance: DNA damage checkpoint

Double strand breaks (DSBs) are among the most toxic DNA lesions which, if left unrepaired, severely compromise cell survival. Chromosomes are particularly susceptible to damage during replication in S phase. Since segregation of damaged chromosomes during mitosis can greatly exacerbate these damages, it is imperative for cells to halt the cell cycle progression and repair the damage prior to the onset of chromosome segregation. DNA damage response (DDR) is a concerted cellular action plan that integrates 1) the network that detects and processes DNA damage 2) the DNA damage checkpoint that halts cell cycle progression and 3) the system that repairs DSBs *via* homologous recombination (HR) or non-homologous end-joining (NHEJ) (Ciccia and Elledge, 2010; Giglia-Mari et al., 2011; Vitor et al., 2020). Many aspects of the DDR are highly conserved across eukaryotic cells and have been studied in detail in both yeast and vertebrate cells (Stracker et al., 2009; Cussiol et al., 2019).

### DNA damage sensing, checkpoint execution and G2 arrest in vertebrate cells

**FIGURE 3:**
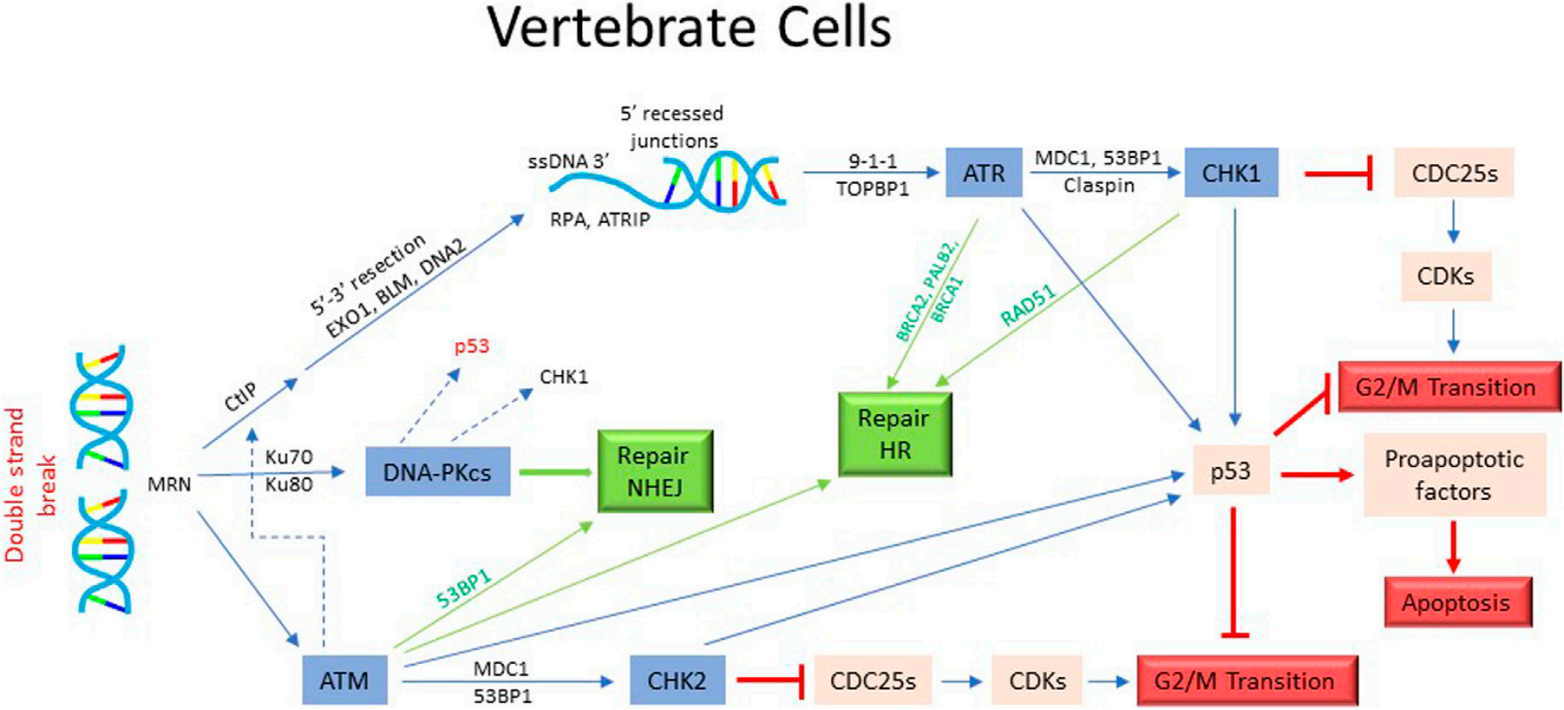
Activation of DNA damage checkpoint and cell cycle arrest in mammalian cells. The DSBs in mammalian cells are recognized by MRN complex and processed with the help of CtIP, EXO1, BLM, and DNA2 proteins to generate 3′ ssDNA extension. This results in the recruitment and activation of ATM/ATR kinase and subsequently, the activation of CHK/CHK2 kinases. The activated kinases then inhibit the effectors required for the onset of mitosis, thus causing cells to arrest in G2. In addition, ATR/ATM/CHK1/CHK2 kinases stabilize p53 which help in the imposition of G2 arrest. P53 also activates apoptotic pathway in certain cellular contexts. As in yeast, the activation of checkpoint effectors in mammalian cells also trigger the DNA repair pathways.

In vertebrate cells, the phosphatidylinositol 3-kinase related protein kinases ATM and ATR, MRN complex, 9-1-1 complex and CHK1/CHK2 are the key elements of the checkpoint activation network. DSB are primarily “sensed/detected” by MRN complex (MRX in yeast) composed of Mre11, Rad50 and Nbs1 (yeast Xrs2) proteins (Tisi et al., 2020; Qiu and Huang, 2021). It carries out initial processing of DSB by generating a short 3′ single strand DNA (ssDNA) overhang (Figure 3). Exo1, a 5′-3′exonuclease, is subsequently recruited for an extensive end-resection to create a long 3′-ssDNA, which is then “coated” by ss-DNA binding factor RPA (Tomimatsu et al., 2017). The RPA-coated ss-DNA is recognized by ATR kinase (Yeast Mec1) *via* its binding partner ATRIP (Ddc2 in yeast). ATR can also be activated by TOPBP1 (yeast Dpb11) recruited at the ssDNA/dsDNA junction (Saldivar et al., 2017). Full activation of ATR also requires recruitment of RAD9-RAD1-HUS1 loader complex (9-1-1 complex: Rad17-Mec3-Ddc1 in yeast) (Delacroix et al., 2007). Another PIKK family kinase ATM (Tel1 in yeast) also contributes to the checkpoint control at G2/M. Transduction of the signal from ATM/ATR to downstream effector kinases involves BRCT-domain containing adaptor proteins 53BP1 and MDC1 and brings these regulators into proximity (Huen and Chen, 2008; Kciuk et al., 2022). CHK1 and CHK2 (yeast Chk1, Rad53) are the main effector kinases activated by ATM/ATR (Figure 3). CHK1 is generally thought to be activated by ATR *via* phosphorylation on S^317^ and S^345^ and CHK2 by ATM *via* phosphorylation on T^68^. However, given the crosstalk between different axes, these phosphorylation-dependencies may not be strict (Smith et al., 2010). All checkpoint kinases phosphorylate and stabilize transcription factor p53, which is involved in cells’ decision to undergo DNA damage-dependent cell cycle arrest, senescence or apoptosis (Oren, 2003; Lavin and Gueven, 2006) (Figure 3).

Once the checkpoint is activated, it targets the mitotic regulators to prevent entry into mitosis. Cdc25C, the phosphatase that plays a critical role in the activation of CDK1, is phosphorylated by ATR, ATM and CHK1 on S^345^ or S^317^ (Liu et al., 2020) (Figure 3). The activation of DNA damage checkpoint also causes phosphorylation of CDC25C on S^287^, creating a 14-3-3 binding site and preventing it from activating CDK1 (Gardino and Yaffe, 2011). CHK1 and CHK2 kinases phosphorylate CDC25C on Ser^216^, causing its proteolytic degradation (Hirao et al., 2000; Gottifredi et al., 2001; Liu et al., 2020). CHK1 also promotes degradation of CDC25A by phosphorylation on Ser^76^ (Jin et al., 2008). In addition, checkpoint activation promotes stabilization of CDK1-inhibiting kinase WEE1 by phosphorylation on Ser^549^ and Ser^287^ residues (Lee et al., 2001). Thus, in mammalian cells, DNA damage checkpoint predominantly targets the members of the CDK1-activation network to prevent entry into mitosis. Inhibition of non-CDK1 kinases such as PLK1 and Aurora A also appear to be important in augmenting DNA damage induced G2 arrest (Smits et al., 2000; Peng, 2013; Joukov and De Nicolo, 2018).

### DNA damage sensing, checkpoint execution and mitotic arrest in *S. cerevisiae*

**FIGURE 4:**
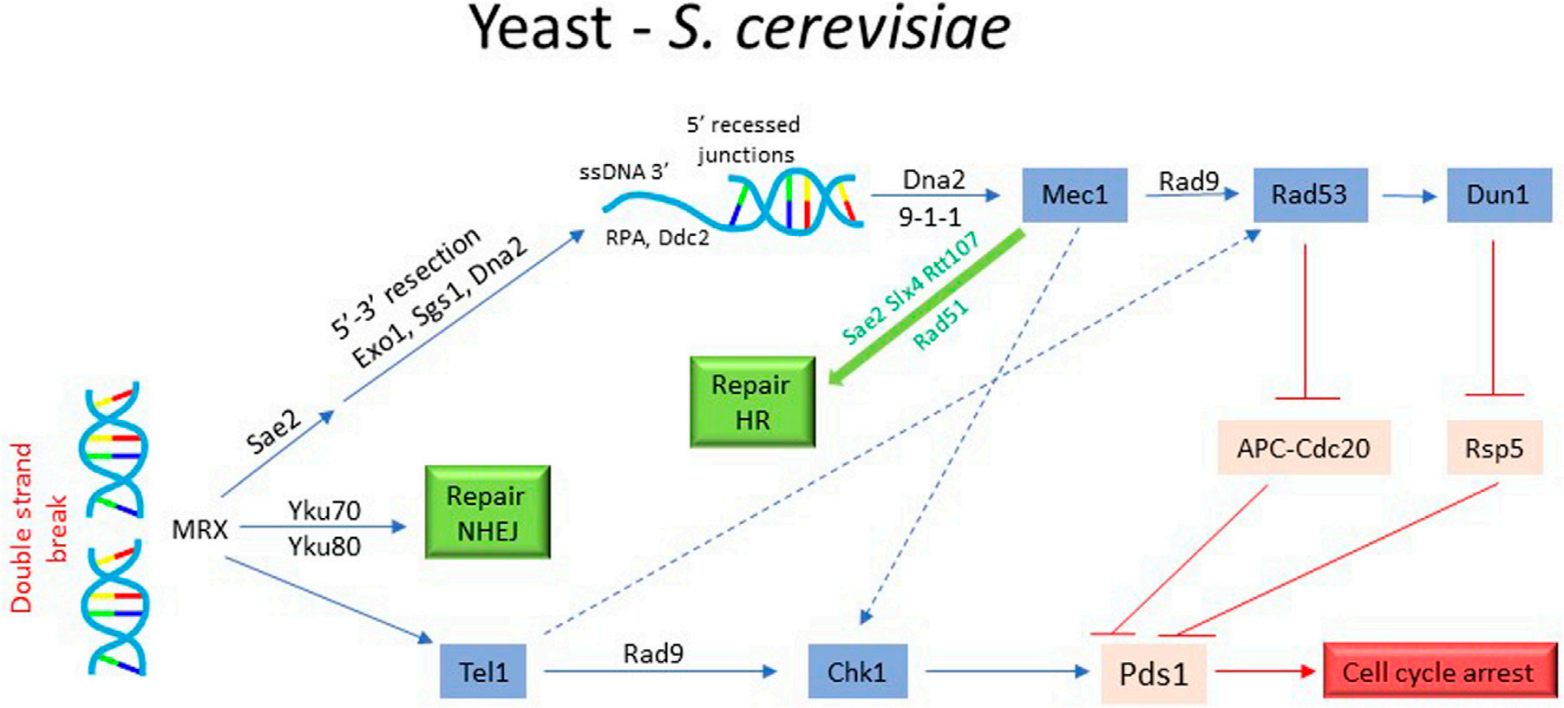
Activation of DNA damage checkpoint and cell cycle arrest in yeast. The activation of DNA damage checkpoint in *S. cerevisiae* requires detection of DSB by MRX complex and its processing by Sae2, Exo1, Sgs1, and Dna2 to generate 3′ ssDNA extension. Subsequently, the recruitment and activation of Mec1/Tel1 leads to the activation of Chk1, RAD53 and Dun1, resulting in the phosphorylation of securin Pds1 and protection from proteolytic degradation by E3 ubiquitin ligases APC^Cdc20^ and Rsp5. The stabilized Pds1 inhibits the separase Esp1 and prevents sister chromatid separation, leading to cell cycle arrest. In parallel, the checkpoint activation also triggers the DNA repair pathways.

Like in vertebrate cells**,** the DNA damage sensing and its initial processing in *S. cerevisiae* is accomplished by the MRX complex (Figure 4). MRX first attracts Tel/ATM to a unresected DSB. Subsequent localization of Mec1 (human ATR) requires end-resection (Lisby et al., 2004; Gobbini et al., 2016). More extensive re-sectioning of the DSB occurs *via* two mechanisms: by Exo1 which removes nucleotides individually from DSB end or by Dna2 endonuclease and Sgs1/BLM helicase (Zhu et al., 2008). Tel1 and Sae2, recruited to the DSB by MRX, initiate Exo1 and Dna2 mediated end-resection (Gobbini et al., 2016; Villa et al., 2016). Cdk1 also plays an important role during resection by phosphorylation and activation of CtIP/Sae2 and Dna2 (Chen et al., 2011; Yu et al., 2019). The localization of Mec1 to the processed DSB (i.e. RPA-coated ss-DNA), is facilitated by Ddc2 (human ATRIP). The adaptor protein Rad9 (mammalian 53BP1) is recruited to the DNA lesions by the scaffold protein Dpb11 (human TOPBP1), which also binds to the 9-1-1 clamp loader *via* Mec1-dependent phosphorylation of Ddc1 (Pfander and Diffley, 2011). Activated Mec1 transmits the damage signal downstream by phosphorylating and activating Rad9 (Figure 4). Mec1-dependent phosphorylation of Rad9 is important for its oligomerization to sustain the damage signal and priming of Rad9 as a scaffold for Rad53/CHK2 localization and for subsequent phosphorylation events (Lanz et al., 2019). Once Rad53 is recruited *via* the docking sites on Rad9, the proximity of multiple Rad53 monomers promotes their autophosphorylation and activation. Activated Rad53 modulates DSB processing by phosphorylating and inhibiting Exo1 (Morin et al., 2008). Mec1 also contributes to the activation of Chk1/CHK1 kinase (Figure 4). While Rad53 is an essential gene for both vegetative growth and DDR in *S cerevisiae*, Chk1 is non-essential for vegetative growth. Chk1 deficient cells exhibit partial defect in G2 arrest in response to ionization radiation (Zachos et al., 2003). Dun1 kinase, a Rad53 paralogue, also features in the dynamics of DNA damage checkpoint (Figure 4). Though structurally similar to Rad53, Dun1 contains a single FHA domain unlike Rad53 that harbors two FHA domains. It interacts with Rad53 through FHA domain and is activated by Rad53-mediated phosphorylation (Bashkirov et al., 2003) (Figure 4). Dun1 is required for DNA damaged-induced transcription of the target genes and the phosphorylation of DNA repair protein Rad55 (Bashkirov et al., 2000; Smolka et al., 2007). It activates the DNA damage-dependent transcriptional program for dNTP synthesis by phosphorylation and degradation of Crt1 and Sml1, both inhibitors of DNA synthesis (Zhao and Rothstein, 2002; Sanvisens et al., 2014). Interestingly, Dun1 deficient cells fail to arrest in response to DNA damage despite the presence of the checkpoint activated Rad53, implying a critical role of Dun1 in DNA damage induced G2/M arrest (Yam et al., 2020) (Figure 4). While the overall scheme of DNA damage checkpoint execution is conserved between yeast and vertebrates, there a few notable differences. For instance, unlike vertebrate cells where tumor suppressor p53 plays an important role in G2 arrest and its cellular outcomes, *S. cerevisiae* lacks p53 homologue or its functional equivalent.

Since G2 phase is extremely brief in *S. cerevisiae* and Tyr^19^ phosphorylation of Cdk1 is necessary but not a rate limiting step for entry into mitosis (Amon et al., 1992; Welburn et al., 2007), the DNA damage checkpoint targets the network that regulate sister chromatid separation. As described above, chromosome segregation in yeast involves cohesion complex, securin Pds1, separase Esp1, APC^Cdc20^ and the spindle apparatus. Mec1-activated Chk1 kinase (and possibly Rad53) phosphorylates Pds1, rendering it resistant to APC^Cdc20^ mediated proteolytic degradation (Cohen-Fix and Koshland, 1997; Wang et al., 2001; Karumbati and Wilson, 2005). There is also some evidence that Rad53 phosphorylates Cdc20, promoting its degradation and thus contributing to mitotic arrest (Wang et al., 2001). This results in the stabilization of Pds1-Esp1 complex, which prevents Esp1-induced cleavage of the cohesin subunit Scc1 and dissolution of chromosome cohesion (Uhlmann et al., 1999). It has been shown that phospho-Pds1 is further protected from E3 ubiquitin ligase Rsp5 by Dun1 (Liang et al., 2013; Yam et al., 2020). These observations clarify Dun1’s role in checkpoint-mediated mitotic arrest. In addition to inhibiting sister chromatid separation, DNA damage checkpoint may also inhibit additional pathways to prevent segregation of damaged chromosomes. It has been proposed that checkpoint maintains APC activator Cdh1 in an active state by inhibiting polo-like kinase Cdc5 (PLK1 in human) to prevent untimely elongation of the mitotic spindle (Zhang et al., 2009).

## Switching-Off the checkpoint: Recovery from G2/M arrest following DNA repair

The checkpoint controls remain active during the repair process and continue to prevent cells from progressing to mitosis. Once the DNA repair is completed, the checkpoint controls must be switched off to permit cells to resume cell cycle progression. Since DNA damage checkpoint is activated mainly by protein phosphorylation events, it is not surprising that phosphatases play an important role in its reversal.

### Recovery from G2/M arrest in vertebrate cells

In mammalian cells, the redundancy within the CDC25 family of phosphatases comes into play during recovery. As CDC25A and Cdc25C are degraded during G2 arrest, the recovery from checkpoint arrest becomes dependent on CDC25B (Bugler et al., 2006; Chen et al., 2021). Another phosphatase WIP1 also increases in abundance several hours after the induction of damage and accumulates at DSBs (Burdova et al., 2019). WIP1 dephosphorylates and deactivates several checkpoint effectors such as ATM, CHK1 and CHK2 (Goloudina et al., 2016). PP1 and PP2A phosphatases also play an important role in deactivation of the checkpoint in that they dephosphorylate γH2A and inactivate ATM-Chk2 axis (Chowdhury et al., 2005; Campos and Clemente-Blanco, 2020). Mitotic regulators such as PLK1 and Aurora A, and proteolytic degradation also play a significant part in the recovery from checkpoint arrest. Suppression of PLK1 does not affect mitotic entry during normal cell cycle but it significantly delays recovery from checkpoint arrest (van Vugt et al., 2004). This delay can be alleviated by depletion of WEE1 suggesting that WEE1 may be the downstream target of PLK1 during checkpoint recovery (van Vugt and Medema, 2005; Kim, 2022). PLK1 also mediates degradation of Claspin leading to disabling of CHK1 (Mailand et al., 2006; Mamely et al., 2006). CHK2 and 53BP1 are also phosphorylated by PLK1 which disrupts their checkpoint function and helps checkpoint recovery (van Vugt et al., 2010; Peng, 2013). Additionally, Aurora A and its cofactor Bora aid cells’ recovery from checkpoint arrest. Since the requirement for Aurora A can be overcome by the expression of activated PLK1, it implies that Aurora A’s role in checkpoint recovery is through the activation of PLK1 (Macurek et al., 2008). Inactivation of p53 by PLK1 may also help cells in the resumption of cell cycle progression (Chen et al., 2006). The Greatwall kinase MASTL has been shown to play an important role in regulating mitotic entry during recovery in human cells (Wong et al., 2016). Similarly, in *Xenopus* egg extracts, Greatwall, together with polo-like kinase Plx1, promotes recovery from checkpoint arrest (Peng et al., 2011).

### Recovery from G2/M arrest in *S. cerevisiae*

**FIGURE 5:**
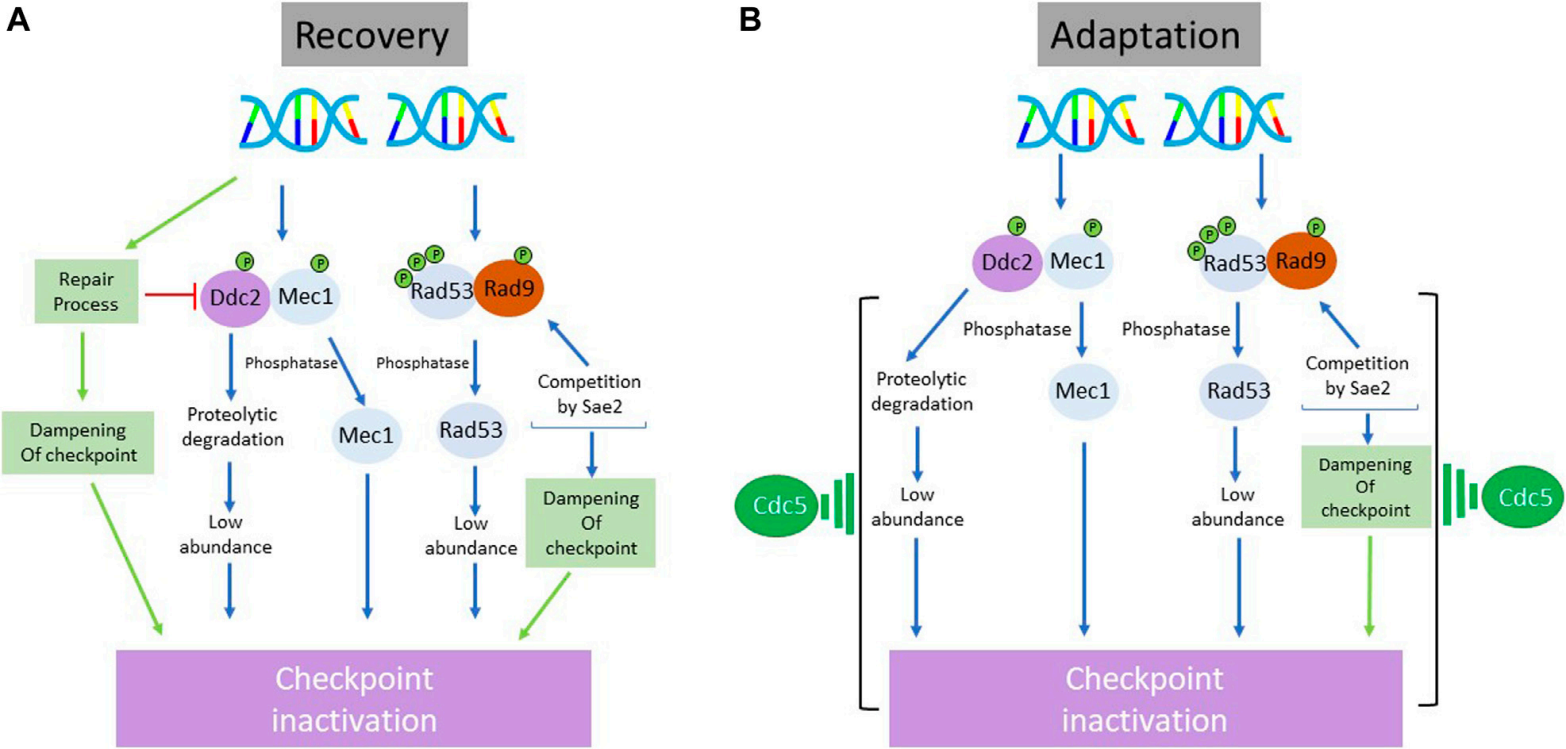
Schematic representation of the processes involved in the inactivation of DNA damage checkpoint during recovery **(A)** and adaptation **(B)** in *S. cerevisiae*. While there is a substantial overlap between the regulatory branches utilized to override the checkpoint-imposed arrest during recovery and adaptation, there are two major distinctions: 1) the involvement of repair machinery in the dampening of checkpoint response during recovery but not during adaptation 2) a prominent role of polo-like kinase Cdc5 in checkpoint inactivation during adaptation but not during recovery. Although Cdc5 is firmly implicated in the execution of adaptive response, the molecular details of its exact role are unclear.

As in vertebrate cells, Ser/Thr phosphatases and proteolytic degradation play an important role in the recovery from G2/M arrest in *S. cerevisiae* (Figure 5A). Since Rad53 is a critical effector in the DNA damage checkpoint, its dephosphorylation has been studied in some detail. PP2A and PP2C classes of phosphatases feature prominently in this context. PP2A Phosphatase Pph3 (and its cofactor Psy2) has been reported to dephosphorylate activated Rad53 and γH2A (O'Neill et al., 2007; Chowdhury et al., 2008; Sun et al., 2011). PP2C phosphatases Ptc2 and Ptc3 (homologues of mammalian WIP1) also dephosphorylate Rad53 after HO endonuclease induced DSB (Guillemain et al., 2007). Ptc2, through its phosphorylation by CK2 on Thr^376^, dephosphorylates the activated Rad53. There is some evidence that the target specificities of Ptc2 and Ptc3 may not completely overlap (Heideker et al., 2007; Gardino and Yaffe, 2011). The action of these phosphatases may remove some but not all phospho-residues from multiply phosphorylated Rad53, suggesting that other recovery specific processes play important roles in full deactivation of the checkpoint. In yeast, checkpoint attenuation begins during repair of the resected DNA. The repair complex Slx4-Rtt107 loads onto the damage sites, displacing the adaptor protein Rad9 (Cussiol et al., 2015). A similar observation has been reported for Sae2 where it competes with Rad9 for binding to the damage site (Yu et al., 2018). This prevents Rad9 from amplifying the damage signal by Rad9 *via* checkpoint kinases. It is speculated that Srs2 might also be involved in removing checkpoint proteins from the damage site during recovery (Dhingra et al., 2021) In addition, proteolytic degradation of Ddc2, the interacting partner of Mec1, may contribute to the dialing down of the checkpoint signaling during recovery. Ddc2 undergoes Mec1-dependent and -independent phosphorylation upon DNA damage and its abundance decreases as cells recover from checkpoint arrest (Paciotti et al., 2000; Memisoglu et al., 2019). A recent report has proposed a positive feedback loop between Ddc2 stability and checkpoint signaling (Memisoglu et al., 2019). Thus, rapid dampening of the checkpoint controls during recovery period requires inputs from multiple effectors.

## Adaptation to DNA damage checkpoint induced arrest: An intriguing cellular behavior

Suspension of cell cycle progression in G2 upon DNA damage and recovery from the arrest following repair of the DNA lesions ensures cells’ survival, fitness, and genomic stability. In the event cells are unable to repair DNA damage, they first arrest in G2/M for a long period but then turn-off the checkpoint controls and proceed to mitosis with damaged chromosomes. Mitotic spindle mediated segregation of damaged chromosomes harms the chromosomes further, thus increasing genomic instability or even causing cell death. This cellular behavior, termed adaptation, is intriguing since cells (particularly unicellular organisms such as yeast) derive no obvious advantage from what may appear to be a “self-destructive action.” A teleological viewpoint is that adaptation allows cells to escape G2 arrest to attempt DNA repair in the subsequent division cycles i.e., “live to fight another day.”

### A brief note on the discovery of adaptation

In 1993, Sandall and Zakian reported that elimination of telomere from a chromosome in *S. cerevisiae* results in Rad9-mediated arrest in G2/M (Sandell and Zakian, 1993). Interestingly, many of these cells recovered from the arrest without repairing the lesion and underwent a few cell divisions, eventually losing this chromosome. A critical study by Toczyski, Galgoczy and Hartwell extended these observations and showed that cells that have suffered a single DSB undergo checkpoint induced arrest in G2/M; however, if the DSB cannot be repaired, cells eventually overcome the checkpoint arrest and proceed to mitosis while the damage is still present (Pellicioli et al., 2001). Toczyski et al. (1997) termed this phenomenon “adaptation” and defined three basic criteria for this cellular behavior: 1) cells halt cell cycle progression because of DNA damage 2) cells eventually override the cell cycle arrest 3) cells still harbor the DNA damage at the time they resume cell cycle progression. Subsequently, it was reported that checkpoint signaling is turned off eventually in *Xenopus* extracts with stalled DNA replication (Yoo et al., 2004). Osteosarcoma cells exposed to ionization radiation (IR) have also been shown to override checkpoint arrest and enter M phase before the damage has been fully repaired (Syljuasen et al., 2006). These studies suggest that cells of multicellular organisms also exhibit “adaptive” behavior when faced with extended checkpoint arrest.

The term “adaptation,” derived from “ad + aptus” meaning “toward + fit,” is a key concept in the context of evolution. It is a process which allows a population of organisms to accumulate advantageous traits and become better suited to its environment (van Vugt and Medema, 2004; Bartek and Lukas, 2007; Leculier and Roux, 2022). With this general concept in view, the term “adaptation” may seem ill-suited for checkpoint deactivation in response to cells’ failure to repair the DNA lesions, as there is little apparent benefit to be gained from this action. It can be argued that switching off the prolonged checkpoint signaling is more akin to desensitization observed in many signaling pathways such as pheromone response in yeast or hormonal response in mammalian cells, which involve feedback inhibition after a significant delay (Jiang and Hao, 2021). Given the transient nature of checkpoint-induced arrest under normal circumstances, it is possible that a deactivation mechanism is built into the checkpoint signaling module. Whether recovery (in repair proficient cells) and adaptation (in repair deficient cells) are mechanistically related events is an important issue.

### Dynamics of adaptation and its mechanistic underpinnings in vertebrate cells

In cancer therapy, apoptosis (after 4–6 h of treatment) following DNA damage by anticancer agents has been considered the main pathway for the eradication of tumor cells. However, this model has been supported specifically by data from cancers of myeloid and lymphoid origin. A substantial body of work had suggested that the treatment sensitivity of many tumor cells to DNA damage-causing anti-cancer therapies is due to their failure to repair the damage and to sustain normal DNA damage response, especially when apoptosis occurs 24–48 h after the treatment and usually after mitosis (Brown and Wilson, 2003). This indirectly hints to the failure of tumor cells to maintain the checkpoint-induced arrest. Indeed, attenuation of checkpoint signaling despite the presence of persistent damage is the central element of the adaptive response. Cells can accomplish this in multiple ways. In vertebrate cells, Claspin acts as an adaptor for the recruitment and activation of CHK1. In *Xenopus laevis*, activated ATR, while propagating the checkpoint signaling, also phosphorylates Claspin at Thr^906^ (Lupardus and Cimprich, 2004; Yoo et al., 2004). This creates a docking site for Plx1 (human PLK1) and allows Plx1 to phosphorylate Claspin on Ser^934^. The modified Claspin then dissociates from the damage-site and undergoes SCFβ-TrCP-mediated degradation (Peschiaroli et al., 2006). These events can result in the dampening of Chk1 activation and checkpoint signal (Mailand et al., 2006). The relationship between CHK1 and PLK1 has also been described in human cells (Tang et al., 2006; Adam et al., 2018). In addition, PLK1 phosphorylates and promotes the degradation of WEE1 which inhibits the CDK1/cyclin B complex (van Vugt and Medema, 2005). In mammalian cells, the level of PLK1 directly affects the cells’ ability to adapt. It has been reported that PLK1 activity continue to rise in G2 arrested cells. When this level reaches a threshold, the cells enter mitosis despite the remaining damage (Liang et al., 2014; Verma et al., 2019). This implies that in mammalian cells, adaptation, instead of being an active surveillance mechanism, is a temporal event determined by the level of PLK1 activity (Wakida et al., 2017).

### Dynamics of adaptation and its mechanistic underpinnings In *S. Cerevisiae*

Adaptation has been extensively studied in *S. cerevisiae*. Repair-deficient yeast cells suffering a single HO endonuclease-induced DSB arrest in G2/M and subsequently, undergo adaptation (Lee et al., 2000; Dotiwala et al., 2013). However, cells with two DSBs remain permanently arrested (Lee et al., 1998). There is evidence to suggest that cells do not respond to the number of DSBs, but to the extent of single stranded DNA produced by processing of the DSBs (Lee et al., 2001). Upon detection of the irreparable DSB, the checkpoint is activated, leading to phosphorylation of Rad53, Chk1, Ddc2, and Cdc20 and cells arrest in G2/M for an extended period (Waterman et al., 2020). Subsequently (∼10 h after the introduction of DSB), Rad53 is dephosphorylated, Ddc2 diminishes in abundance and cells harboring the DSB proceed to mitosis (Memisoglu et al., 2019). It is noteworthy that having undergone adaptation once, yeast cells continue to divide without reactivating the checkpoint in the subsequent rounds of division cycles. As repeated divisions cause further accumulation of chromosome aberrations, most of these cells eventually lose viability. IR-exposed human osteosarcoma cells undergoing adaptation also accumulate chromosome aberrations (Kalsbeek and Golsteyn, 2017), implying that checkpoint adaptation response in mammalian cells increases the risk of accumulating genetically abnormal cells that could potentially undergo malignant transformation.

An early genetic screen in yeast to identify genes involved in adaptation to HO-induced single DSB yielded yeast polo-like kinase *CDC5* and *CKB2*, a non-essential subunit of casein kinase II (Toczyski et al., 1997). Subsequent studies identified additional genes that play a role in checkpoint adaptation, namely, phosphatase Ptc2 and Ptc3 (Leroy et al., 2003), telomeric Ku complex subunits Yku70 and Yku80 (Lee et al., 1998), helicase Srs2 and DNA-dependent ATPase Tid1 (Ferrari et al., 2013; Bronstein et al., 2018). Ptc2 and Ptc3 dephosphorylate Rad53 to terminate checkpoint signaling (Figure 5B). Phosphorylated by Casein kinase II on Thr^376^ located in a TXXD motif, Ptc2 can bind to the FHA1 domain of Rad53 and facilitate dephosphorylation of Rad53. Consistent with this, *ptc2Δ ptc3Δ* double mutant exhibits normal cell cycle kinetics but fail to undergo adaptation (Guillemain et al., 2007). In yku80 mutant, Ku DNA binding complex is disrupted and resection is accelerated; consequently, cells become permanently arrested in G2/M (Clerici et al., 2008). Of all the genes influencing adaptive response, Cdc5 kinase has garnered much attention. DNA damaged cells harboring adaptation-defective *cdc5-ad* allele remain permanently arrested in G2/M, even though Cdc5-ad retains its kinase activity (Toczyski et al., 1997; Rawal et al., 2016). Cdc5 overexpression, on the other hand, accelerates adaptation and partially suppresses other adaptation defective mutants (Donnianni et al., 2010; Vidanes et al., 2010). In Cdc5 overexpressing cells, though the early steps of checkpoint activation such as recruitment of Ddc1/Ddc2 and Mec1 activation remain unaffected (Donnianni et al., 2010), Rad53 hyperphosphorylation is conspicuously reduced (Vidanes et al., 2010). The mechanism by which Cdc5 kinase diminishes the hyperphosphorylation of Rad53 kinase is not clear. Cdc5 can phosphorylate Rad53 and this modification appears to be important for adaptation (Schleker et al., 2010). It is possible that Cdc5-mediated phosphorylation of Rad53 triggers its deactivation. However, it is uncertain if Rad53 dephosphorylation is the main factor in promoting adaptation since two other mutants *cdc5-16* and *cdc5* ^*T238A*^ remain arrested after Rad53 is dephosphorylated (Ratsima et al., 2016; Rawal et al., 2016). In yeast, the Mec1-Ddc2 complex localizes to the damage-site, triggering the recruitment and activation of Rad53. Turning off the checkpoint during adaptation may also involve these upstream dynamics. There is evidence to suggest that Mec1 is phosphorylated on S^1964^ residue and that the activated Ddc2 is degraded during adaptation (Memisoglu et al., 2019). These events will limit Mec1 localization to the break site and consequently, attenuate the checkpoint signaling cascade (Bandhu et al., 2014).

Cells carrying uncapped telomeres also exhibit checkpoint activation and adaptation. Single strand DNA present at the telomeres is normally capped by CST (Cdc13-Stn1-Ten1) and Ku (yKu70/yKu80) complexes (Westmoreland et al., 2018), that prevent activation of DNA damage signaling. The yeast ts mutant *cdc13-1* is defective in telomere capping, which results in an extensive resection by Exo1 (Langston et al., 2020), leading to the activation of DNA damage-checkpoint signaling and G2/M arrest. Just as in the case of cells harboring HO-induced single DSB, *cdc13-1* cells undergo adaptation involving Cdc5 and CK2 and undergo accelerated adaptation in response to Cdc5 overexpression (Ratsima et al., 2016; Coutelier et al., 2018).

## Recovery and adaptation: Same exit different doors?

A release from the arrest-state and resumption of cell cycle progression are the phenotypic outcome of both recovery and adaptation. In both cases, the checkpoint signaling is turned off and mitotic machinery is re-engaged; however, the cellular contexts are very different. While cells recovering from G2 arrest do so after the DNA damage has been repaired, cells undergoing adaptation override arrest when the damage is still present. As discussed above, during the repair-driven recovery, checkpoint signaling is dampened in step with the DNA repair. However, terminating the upstream signals is not sufficient for recovery; the protein modifications of checkpoint effectors present during G2 arrest must also be reversed. In mammalian cells, WIP1, PP1 and PP2A dephosphorylate γH2A and inactivate ATM-Chk2 axis (Ramos et al., 2019). In yeast, phosphorylated Rad53 is a prominent player in the checkpoint signaling and is dephosphorylated by Ptc2/Ptc3 phosphatases during recovery (Leroy et al., 2003). In addition, the regulators of mitosis play an important role in the process of recovery. Although polo-like kinase PLK1 or CDC25B phosphatase are not strictly required for mitotic entry in undamaged cells, both these regulators are important for the recovery from DNA damage-induced G2 arrest (van Vugt et al., 2005; Bansal and Lazo, 2007; Hyun et al., 2014). PLK1 also promotes proteolytic degradation of the CDK1 inhibitory kinase WEE1 (van Vugt et al., 2004).

The adaptive response shares some regulatory aspects with the recovery process. Most prominent in this context is the role of phosphatases: WIP1, PP1, and PP2A in mammalian cells and Ptc2/Ptc3 in yeast (Heideker et al., 2007; Freeman and Monteiro, 2010). It is not clear, at least in yeast, whether Ptc2/Ptc3 phosphatases act constitutively or require adaptation-specific activation. Since the option of dampening down of upstream events by repair complexes (during recovery in yeast) is not available during adaptation, limiting the continued activation of these events by other means is important (Mailand et al., 2006). In mammalian cells, involvement of polo-like kinase is another element shared by both recovery and adaptation response (Liang et al., 2014). PLK1 can phosphorylate and promote the degradation of WEE1 during both responses (Takaki et al., 2008). However, the role of polo-like kinase Cdc5 in adaptation in yeast is somewhat perplexing. That adaptation defective (and repair proficient) *cdc5-ad* mutant can efficiently recover from DNA damage-induced arrest implies that Cdc5 is not required for recovery (Pellicioli et al., 2001; Vidanes et al., 2010). The fact that both *cdc5-ad* and *cdc5Δ* mutants are adaptation deficient and overexpression of Cdc5 accelerates adaptation suggests that Cdc5 is a key rate limiting factor for the adaptative process (Shaltiel et al., 2015; Serrano and D'Amours, 2014). As dephosphorylation of Rad53 is one of the prominent features of cells undergoing adaptation, it is not clear how Cdc5 causes dephosphorylation of Rad53. It has been reported that Cdc5 does not inhibit the formation of Rad9-Rad53 complex but does prevent hyperphosphorylation of Rad53 (Vidanes et al., 2010). Since Rad53 is proposed to prevent BRCT-SCD domain-specific oligomerization of Rad9 required to maintain checkpoint signaling (Usui et al., 2009), it is possible that this action of Rad53 limits its own activation. As Cdc5 also inhibit Rad53 autophosphorylation *in vivo*, this may result in the enhancement of the negative feedback loop between Rad9 and Rad53 (Usui et al., 2009; Lopez-Mosqueda et al., 2010; Vidanes et al., 2010). As Rad53 has been reported to inhibit Cdc5 in response to DNA damage (Sanchez et al., 1999; Coutelier et al., 2021), this can potentially add an additional regulatory branch. Important as dephosphorylation of Rad53 during adaptation is, it remains unclear if this is the primary event that initiates adaptation. Recently, a change in the abundance of Mec1-associated protein Ddc2 is suggested to be an important event in adaptation in response to a persistent DSB (Memisoglu et al., 2019).

## Closing remarks

Halting of cell cycle progression by checkpoint mechanism in response to DNA damage is a critical aspect of survival for a dividing cell. Cells utilize various means to temporarily decouple from the division protocol to execute the repair program. Reengagement of the division machinery after this transient hiatus is equally important and requires disconnection from the checkpoint protocol. The nature of this reengagement and its outcome is dependent on the execution of repair program: successful repair maintains genomic stability, enhances viability (recovery) and results in healthy progeny, whereas failure to repair results in genomic instability and loss of viability (adaptation). Checkpoint needs to be extinguished during both recovery and adaptation. In organisms as distantly related as yeast and human, recovery and adaptation involve some common strategies and regulatory elements to extinguish the checkpoint (Figure 5). However, there are some notable differences. Since the repair process itself acts to diminish the checkpoint signaling, DNA repair and recovery are closely coupled events. In repair-deficient (or inefficient) cells, the repair mediated whittling down of checkpoint signaling is not an option available during adaptive response. Therefore, cells must achieve it by employing other strategies in which Polo-like kinase plays a critical role as described in the previous sections. Importantly, it is unclear when cells ‘decide’ to give up attempting to repair the DNA damage and to initiate the adaptative process. Based on the available evidence, adaptation does not appear to be an active process. Rather, it may be a timed response in that the checkpoint erosion is naturally coupled to its activation. Unless aided by the DNA repair system, the natural checkpoint inactivation is perhaps a slow process requiring progressive accumulation of some effectors or establishment of some feedback loops. This would explain why adaptive response is so prolonged an event. Nevertheless, as cell cycle reentry in the presence of DNA damage (as in adaptation) is a harmful undertaking resulting in genomic instability, adaptive response may be relevant to cancer progression in multicellular organisms. Upregulation of proteins implicated in recovery/adaptation is reported in many cancers and is correlated to poor treatment outcomes. A deeper understanding of the regulatory interfaces between DNA damage/repair/checkpoint controls/recovery/adaptation would be relevant to cancer prevention and treatment.
